# Precursor‐Derived Sensing Interdigitated Electrode Microstructures Based on Platinum and Nano Porous Carbon

**DOI:** 10.1002/open.202400179

**Published:** 2024-08-19

**Authors:** Lukas Mielewczyk, Lydia Galle, Nick Niese, Julia Grothe, Stefan Kaskel

**Affiliations:** ^1^ Inorganic Chemistry I Technische Universität Dresden Bergstraße 66 01069 Dresden

**Keywords:** Platinum, Carbon, Microsensor, Nanoimprint Lithography, Piezoelectric Inkjet Printing

## Abstract

Interdigital electrodes were prepared using nanoimprint lithography and piezoelectric inkjet printing. These processes are simpler and more cost‐effective than the industrially used electron beam lithography because of their purely mechanical process step. For the investigation of material dependence, platinum as well as carbon electrodes were fabricated. Afterwards electrodes with various line widths and spacings were tested for the application as a chemiresistive sensor for ferrocenyl‐methanol and the influence of the gap‐width and conductor cross‐section on the sensitivity was investigated. The general suitability of the systems for the production of such structures could be confirmed. Structures with a limit of detection (LOD) down to 1.2 μM and 35.9 μM could be produced for carbon and platinum, respectively, as well as a response time of 3.6 s was achieved.

## Graphical abstract

This work shows insights in the preparation of patterned micro sensors prepared by nanoimprint lithography and piezoelectric inkjet printing. Therefore two different materials namely platinum and carbon were compared relating the limit of detection, sensititvity and response time. Various line widths were tested for the usage as a chemiresistive sensor for ferrocenyl‐methanol.

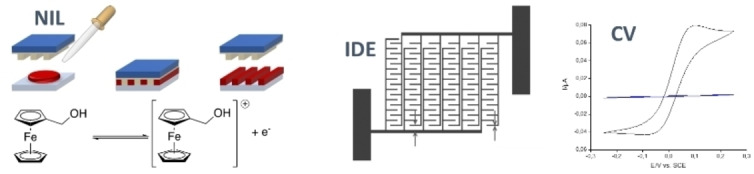

## Introduction

As industrial and commercial demand for small, thin, efficient and portable electronic devices increases, the demands for new and cost‐effective production techniques of structured electrodes increase too.[^1^, ^2^, ^3^, ^4^, ^5^] Next to many others two dimensional electrodes^[6]^ and interdigitated electrodes play a big role in this development. Because of their advantages, interdigitated microelectrodes are key components in electronics. Such structures are used in a wide range of applications such as supercapacitors or in touch‐screen‐technology.[^7^, ^8^, ^9^, ^10^, ^11^] In the field of micro‐supercapacitors such structures are already realized and miniaturized down to a linewidth of 40 μm or less showing excellent performance.[^11^, ^12^] Similarly also interdigital electrode based sensors have an outstanding potential to serve as thin and ultra‐high sensitive sensor devices (*e. g*. for the control of technical processes, environmental monitoring, healthcare).[^1^, ^2^, ^8^, ^13^, ^14^, ^15^, ^16^] Due to their miniaturized patterns down to few nm in diameter, the structures have a higher surface‐to‐volume ratio than macroscopic structures and also very short diffusion paths. These features result in an overall high performance and sensitivity. Latime *et al*. already calculated the dependence of the capacitance of such electrodes on their lateral dimensions and particularly on the gap width.^[4]^ The smaller the dimensions get, the more difficult the explanation of mechanisms can get. Not only geometries but also material design and the conductor cross section come into play. For efficient sensing, the control and knowledge about all parameter influences is crucial. Furthermore, all parameters should be adapted to the respective application since different requirements also entail optimization of other parameters.

For the fabrication of structured interdigital electrodes various methods are reported and used in literature.[^17^, ^18^] Methods like photolithography or screen‐printing however have disadvantages concerning the number of production steps causing high production costs.[^17^, ^19^] In comparison such methods still show better resolutions and precision than most other techniques.[^5^, ^20^] To overcome these limitations and to allow for continuous fabrication processes (*e. g*. roll‐to‐roll), nanoimprint lithography (NIL) is considered to be one of the most promising alternatives.[^17^, ^21^] Here much easier production opens the way for testing several materials and modelling of the resulting structures in an more effective way. In this procedure, a stamp made of a soft polymer, typically cross‐linked polydimethylsiloxane (PDMS), is used for the printing of any desired structure with very high resolution and reproducibility. The actual structuring process is purely mechanical and is carried out with the aid of a printer in only one process‐step.[^22^, ^23^, ^24^]

Recently, three‐dimensional (3D) printing processes play a crucial role for the production of micro‐patterned structures due to their huge variety in terms of design and geometry.[^25^, ^26^] Creating such complex three‐dimensional designs is possible using a layer‐by‐layer method. With this established technique, resolutions from 5 to 200 μm can be achieved.^[27]^ 3D‐printing processes are nowadays commonly used in the field of architecture,^[28]^ biomechanicals,^[29]^ energy storage[^30^, ^31^] and prototyping.^[29]^

There are also many possibilities in the choice of materials for specific applications. For example various inks and different processes for many noble metals such as gold, silver and palladium are very present for sensing materials.[^32^, ^33^, ^34^] Nobel metals are highly conductive and furthermore, they are inert to many sensing media, especially when transferred to biosensing applications. Also, carbon is a very common material in supercap and battery applications and could also serve for highly efficient sensor materials, especially in iontronics and biosensing.^[35]^

Here, we report the comparison of two different printing techniques as well as two different sensor materials in application of a liquid model analyte system. We show the influence of various geometry parameters addressing different IDE‐resolutions and line thicknesses with the two methods (piezoelectric inkjet printing and NIL). We also report here the strong dependence of sensor material and porosity on the sensing performance, as we compare a platinum based and a porous carbon‐based sensor in the detection of ferrocenyl‐methanol in a 2‐electrode setup. Ultimately, we give insights into the possibilities to influence various actuating variables of a micro sensor.

## Experimental Section

### Platinum Precursor

For the complex 315.8 mg (0.94 mmol) Pt(NO_3_)_2_ (Chempur) were suspended in 20 ml of acetonitrile (99.9 %, Chempur). Afterwards the suspension was heated to reflux for 1 h. After cooling to room temperature, the solvent was removed under vacuum, yielding an orange powder.^[36]^ For the synthesis of the platinum precursor bis(acetonitrile)dinitratoplatinum(II) (Pt(ACN)_2_(NO_3_)_2_) was dissolved with a ratio of 2 : 3 in dimethyl sulfoxide (DMSO, 100 %, VWR chemicals) under ultrasonification.

### Carbon Precursor

For the synthesis of the carbon precursor 0.15 g (1.53 mmol) sulfuric acid (95 %, VWR) were dissolved in 4.94 ml (274.21 mmol) deionized water. Further 1.5 g (4.38 mmol) sucrose (>99 %, AppliChem) were dissolved in the mixture using ultrasonification. Finally, 1.26 ml (21.86 mmol) Ethanol (99 %, VWR) were added dropwise under stirring.

### Nanoimprint Lithography

To realize the patterned structure on the substrates with solvent assisted nanoimprint lithography a Micro‐Contact‐Printing System *μ‐CP 3.0* from *GeSiM mbH* was used. Additionally, PDMS stamps (Sylgard 184 elastomer kit, *Dow Chemicals*) were used, the preparation is described elsewhere.^[34]^ For the printing, glass substrates (Corning 1737, DELTA Technologies) (25 mm×25 mm×1.1 mm) were used. They were prepared in a piranha solution (1 part 30 % H_2_O_2_, 3 parts conc. H_2_SO_4_) for at least 1 h. Afterwards, they were cleaned with deionized water and ethanol and dried in a nitrogen stream. Prior to the printing process the substrates were activated with cold‐inductive coupled argon plasma, which was generated with a plasma finger *KNIPEN* of the company *Neoplas Tools*. The stamp was pressed in a 2.5 μl droplet of the ink on top of the substrate. The ink was then cured at 393 K for 15 min and after the stamp was removed.

For the piezoelectric inkjet printing of the precursors a *BioScaffolder 3.2* and a piezoelectric micropipetting tip *Nano‐Tip J* from *GeSiM mbH* was used. For this, the same substrates were used as described previously. The printing procedure with this precursor was described elsewhere.^[37]^ Briefly, the glass substrates were heated to 80 °C before printing. The used tip produced drops with a volume of around 400 pL which result from an applied voltage which was modulated by pulse width and a certain frequency (150 Hz, 90 μs, 55 V). Then the drops were placed on the substrate in continuous lines.

### Platinum Formation

The pyrolysis of the printed structures was carried out at 523 K and 873 K for 2 h each under air with a constant heating rate of 60 K h^−1^.

### Carbon Formation

After printing, the IDEs were stored in a drying oven at 393 K until the actual pyrolysis. Therefore, the printed structures were heated to 1173 K for 2 h under argon with a heating rate of 150 K h^−1^.

### Preparation of the Micro Sensors

After pyrolysis both types of printed structures were cleaned with deionized water and dried in a nitrogen flow. To contact the electrodes, a carbon‐based conductive lacquer *Electro DAG* was applied and dried at 353 K for at least 30 min. For the electrochemical measurements a 200 μl reservoir made of ePTFE (expanded polytetrafluorethylene) was glued to the substrate.

### Electrochemical Testing

The electrochemical testing was carried out with a *VMP3‐potentiostat* from *BioLogic Instruments*. Cyclic voltammetry (CV) was recorded from −0.5 −1 V as well as −0.3–0.3 V at 10 mVs^−1^ against an internal standard SCE electrode with a 100 mM KCl in phosphate buffer solution including 1 mM ferrocenyl‐methanol. The electrolyte was applied in a prepared pool right before the measurement. Potentiostatic electrochemical impedance spectroscopy (PEIS) was carried out in a frequency range from 5 MHz to 100 kHz to measure the equivalent series resistance (ESR). For chronoamperometric (CA) measurements the potential of the peak current from the oxidation of ferrocene methanol was used. For the measurement a series of dilutions of ferrocenyl‐methanol was used with the concentrations of 1000, 500, 250, 125 and 62.5 μM in the before mentioned buffer solution. Each measurement lasted 180 s.

**Figure 1:**
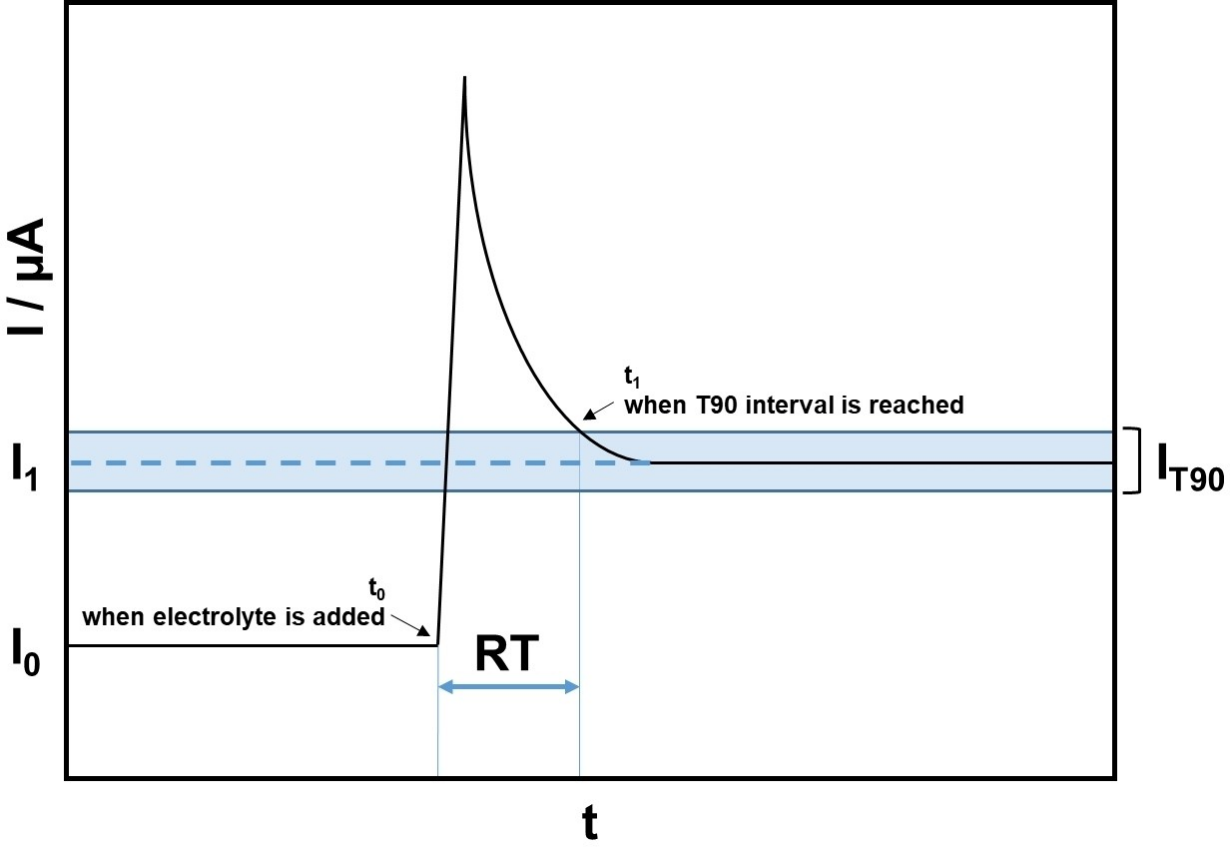
Principal of the T90 test applied on a schematic CA measurement. Here, t_0_ indicates the addition of the electrolyte and t_1_ shows the time where the measured value reaches the ± 10 % interval of the expected value. The difference of t_0_ and t_1_ is defined as the response time RT.

Further, the response time was determined by performing a CA with 50 μl buffer‐solution. After 100 s the same volume of 1 mM ferrocene methanol solution was added. From this point, the time until the measured value reaches the expected value with a variance of ± 10 % is defined as the response time shown in Figure 1.

## Results and Discussion

**Figure 2:**
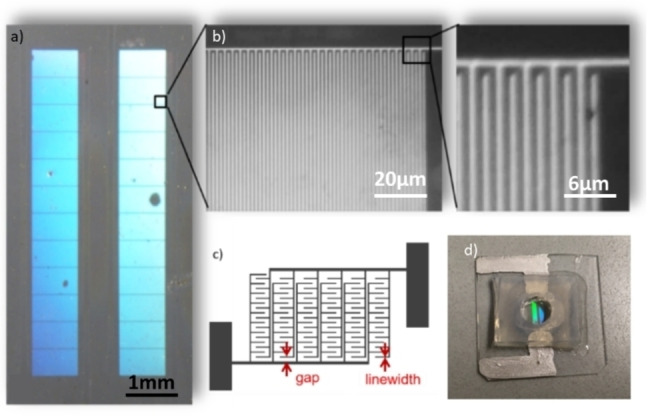
a) Microscope image of a printed electrode (0.5 μm linewidth), b) magnification 20‐times and 80‐times and c) scheme of an IDE and d) an assembled device before testing.

Here we report the preparation of high‐performance sensors based on two precursor systems with a very cost‐effective printing method with comparable sensitivity to commercially available sensors (see Figure 2). To investigate the performance as a sensor, ferrocenyl‐methanol served as a relatively simple model analyte system because it has been intensively studied in literature. As another key element for downsizing the sensing device, we will show, that the design of those interdigital electrodes allows them to be operated in a two‐electrode setup. Thereby no additional reference electrode is required, and the system is working effectively in a very simple arrangement.

The preparation of the micro sensors requires multiple steps to take. At first suitable precursors for both platinum and carbon need to be found. They need to fulfil the requirements for the printing process of NIL as well as the piezoelectric inkjet printing with respect to viscosity and shape‐stability. Most significant, after printing of the precursors, a spatial separation of the electrode lines must be ensured without any residual platinum or rather carbon species between them. Nevertheless, the lines must be stable enough to avoid cracks, which eventually might lead to a loss of conductivity. Therefore, the material itself needs to show a high conductivity and the potential to detect redox reactions. For the application of achieving high‐resolution patterns two literature known precursors are used. Namely a platinum precursor consisting of bis(acetonitrile)‐dinitratoplatinum(II) dissolved in DMSO^[36]^ is used as well as a sucrose carbon precursor which undergoes a condensation‐cross‐linking catalysed by sulfuric acid.^[11]^

### Platinum Interdigital Electrodes

**Figure 3:**
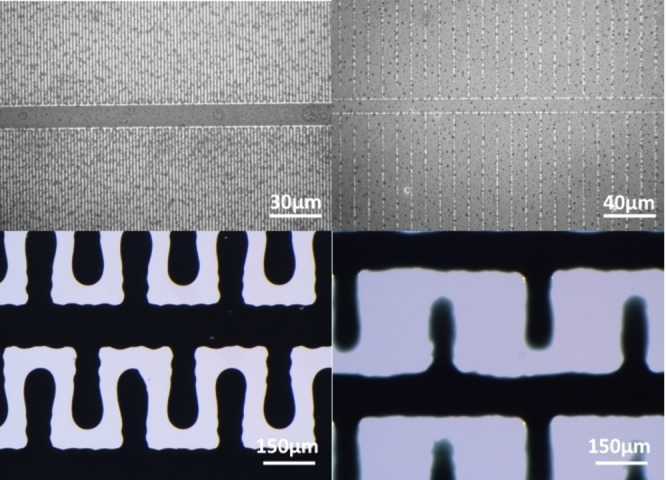
Microscopic images of the used structures a) IDE1, b) IDE2, c) IDE3C and d) IDE4C.

To investigate the influence of the geometry of the interdigital electrodes we realized IDEs with different line widths and distances shown in Table 1 and additionally depicted for all structures in Figure 3.

**Table 1:** Summary of the geometries of the platinum IDEs with the resulting resistance, LOD and sensitivity.

	Method	Linewidth [μm]	Line distance [μm]	Surface area [mm^2^]^[11]^	ESR [kΩ ]	LOD [μM]	Sensitivity [μA/mM]
IDE1	NIL	0.5	1	6.7	26.3	76.9	2.02*10−2
IDE2	NIL	1	10	7.7	18.7	118.9	1.08*10−2
IDE4	Inkjet	100	1200	8.9	11.0	35.9	7.20*10−5

**Figure 4:**
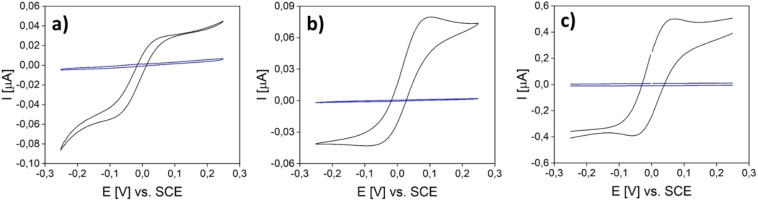
CV curves of a) IDE1 b) IDE2 and c) IDE4 with buffer solution (blue) and ferrocene methanol (black) as analyte.

To prove the functionality of the platinum electrodes, CVs (Figure 4) in buffer solution as well as in a 1 mM ferrocenyl‐methanol solution were measured. The model analyte is used in this case because of its capability of performing a reversible oxidation of the iron centre from Fe^2+^ to Fe^3+^ without any decomposition, when voltage is applied.^[38]^ The redox reaction results in a current flow which can be detected. Independently of the IDEs’ dimensions, all IDEs show hardly any current flow in the neat phosphate buffer at pH 7. The absence of any obvious electric current in this potential range confirms the complete isolation of the two fingers of all electrodes. Thus, there is no continuous platinum film acting as an electrical contact between two neighbouring fingers of the same IDE. Otherwise, such an electrical contact would cause a short circuit and would give rise to linear increase of the current as a function of the applied potential. The comparison of both measurements shows a huge increase of the measured current for the added ferrocene methanol solution as well as the typical redox peaks. For further investigations, the oxidation peak at around 0.1 V was used.

The electrochemical measurements show that the prepared sensors are suitable for the chosen redox system. To determine the LODs of the structures, CA were performed with the ferrocenyl‐methanol concentrations mentioned above. For the smallest IDE structure IDE1 a LOD of 76.9 μM (Figure S1.) was determined. In comparison to this, the IDE2 structure (Figure S2) shows a LOD of 118.9 μM which is quite higher. Although the geometric surface area is nearly the same, the ten times increased diffusion length is mostly responsible for the increased LOD. The opposite trend is observed in the ESR (Figure S1., Figure S2.) which decreases from IDE1 to IDE2 from 26.3 to 18.7 kΩ , which might result from the increasing conductor cross‐section. The same effect is also demonstrated for the IDE4 structure where the cross‐section diameter increases significantly and the ESR (Figure S3.) decreases to 11.0 kΩ simultaneously. This and a nearly doubled surface area in comparison of IDE2 results in a significant lower LOD of 35.9 μM, although the diffusion length is more than 100‐times longer. Another important aspect to consider is the sensitivity of the prepared sensors. The measurement shows that the sensitivites of IDE1 and IDE2 are similar with values of 2.024*10^−2^ μA/mM and 1.08*10^−2^ μA/mM. However, the largest structure IDE4 shows a lower sensitivity of 7.2*10^−5^ μA/mM which indicates that a much smaller change in the value of the input variable leads to a noticeable change in the value of the output variable of the sensor. A general aspect which influences all values measured is the high risk of defects. The smaller the structures the more defects can occur, which mostly affects the LOD and sensitivity due to the unused fingers of the IDE structures. When they are not connected to the conductive structures, they are not available for the detection of the analyte. This phenomenon only becomes significant for the very thin structures of the NIL‐printed sensors.

### Carbon Interdigital Electrodes

**Figure 5:**
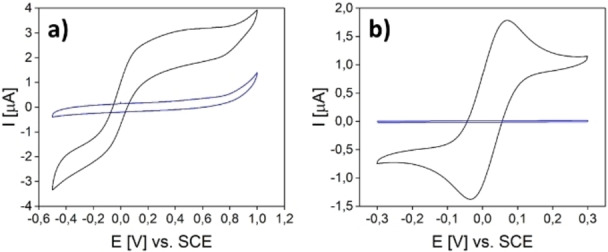
CV curves of a) IDE3C and b) IDE4C with buffer solution (blue) and ferrocene methanol (black) as analyte.

The procedure used for the electrochemical measurements is the same as for platinum IDEs. The geometries of the used IDEs as well as the measured values are summarized below in Table 2. Similar to platinum IDEs, the carbon IDEs are equally useful for the chosen redox system (see Figure 5). In the same manner as before, the LODs of the structures (Figure S4., Figure S5.) were determined with 64.6 μM and 1.2 μM for IDE3C and IDE4C. As mentioned, it is expected that the diffusion length as well as the surface area have the most influence on the performance of the sensors.

**Table 2:** Summary of the geometries of the carbon IDEs with the resulting resistance, LOD and sensitivity.

	Method	Linewidth [μm]	Line distance [μm]	Surface area [mm^2^]	ESR [kΩ ]	LOD [μM]	Sensitivity [μA/mM]
IDE3C	Inkjet	100	600	8.9	3.2	64.6	4.0*10−8
IDE4C	Inkjet	100	1200	14.1	0.9	1.2	2.42*10−7

Even though the diffusion length of the IDE4C is two times higher, the LOD decreases significantly. These results suggest, that the influence of the geometric area is stronger in comparison to the diffusion length. In contrast to this, the sensitivity might be more influenced by the diffusion length compared to the surface area. Out of both IDE3C showed a smaller sensitivity of 4.0*10^−8^ μA/mM compared to 2.42*10^−7^ μA/mM of IDE4C. The results for the ESR show a nearly similar value for IDE3C and IDE4C with 3.2 kΩ respectively 0.9 kΩ which can be explained by the similar expected conductor cross‐section of the structures.

In direct comparison of the IDE4 structures made of platinum and carbon the carbon IDE showed an overall preferable performance. Especially the LOD of 1.2 μM for the carbon structure is much better than 35.9 μM from the platinum structure. Additionally, the ESR of the carbon IDE is preferable with 0.9 kΩ in comparison to the 11.0 kΩ from the platinum IDE.

This applies also for the sensitivity which is lower for carbon than for platinum with 2.42*10^‐7^ μA/mM respectively 7.2*10^−5^ μA/mM. On one hand the reason for this might be found in the surface area which was calculated without considering the porosity of the carbon material. Platinum has a smooth surface compared to the carbon material. During the preparation of platinum, the precursor is decomposed followed by sintering of the metal particles resulting in this smooth surface. Carbon on the other side does not undergo this sintering step. During decomposition of the precursor pores evolve in the material resulting in a porous structure. It is literature known^[11]^ that the used carbon material has a specific surface area of 735 m^2^/g which increases the electrode surface area in comparison to the calculated geometric surface area. However, the real surface area of the printed structures could not be determined. On the other hand, one would expect a lower resistance for platinum than for carbon which might connect in this case to a defective structure derived from platinum in comparison to the carbon structures and the much lower cross‐section of the platinum fingers. When comparing both methods regarding a further improvement of the mentioned properties, NIL is restricted in the controlling of the thickness which could influence the sensitivity and LOD. The beforehand used mask for the preparation of the stamps defines a certain value for the thickness. However, it is strongly dependent on the precursor's viscosity to which degree these gaps are filled. For low viscosities it cannot be ensured that the gaps are completely filled due to low capillary forces. Further precursor systems with too high viscosity could have to high surface tension which also hinders the filling of the gaps of the stamp. The piezoelectric inkjet printing also has high requirements concerning the viscosity. The formation of the droplets as well as the stability of the droplets on the glass surface are highly dependent in the viscosity. However, when the precursor has optimised condition, it is possible to print multiple layers over each other.

An obvious advantage of the inkjet printing is the high versatility. Here, it is possible to do fast adjustments on the geometry which is desired for the IDE. NIL is not this versatile since it needs masks for the preparation of the stamps which are still be produced by photolithographic methods. However, this allows for the production of IDEs with lower line widths since the masks are produced with photolithography. The inkjet printing is limited in terms of downsizing because the line width is highly dependent on the size of the droplets, which are in a range of around 200 μm. Concluding this, both methods have certain advantages and disadvantages, but in combination they complement each other by covering a wide range of sizes and potential precursors.

### Response Time

To determine the response time, which the sensor takes to react to a change in the system the T90 test was used. Therefore, CA was performed with 50 μl buffer solution. After the measured current stays at a constant value, the same amount of 1 mM ferrocenyl‐methanol solution was added. An overshooting peak will occur immediately which will eventually approach the new current value. As soon as the measured current reaches this value with a variance of 10 % the response time can be determined. The measured times for the prepared IDE structures are summarised in Table 3. It is expected that the response time is strongly dependent on the diffusion length between the printed fingers of the structures. Therefore, the smallest structure should show the shortest response time. The results cannot confirm this with a value 9.8 s for the smallest structure IDE1. The reason for this might be the high number of defects in the small structure which virtually increases the distance between two conductive fingers within the structure. Also, the ESR might impact the response time to a certain level having an electric rather than a geometric influence. By creating larger structures, the rate of defects significantly decreases which also decreases the response time for the IDE2 structure to 3.6 s. When creating even larger structures the response time increases as expected for IDE3 to 11.3 s and for IDE4 to 17.9 s and 22.6 s for carbon respectively platinum. The IDE4 shows a slight difference of the response time for the different materials. Here the carbon IDE shows a 5 s faster response in comparison to the corresponding platinum IDE. The difference might be found in the higher surface area as well as the higher conductivity of the carbon material resulting in overall preferable performance of the carbon sensors. Compared to other literature known liquid phase sensors the response time is in a similar range, independent from the analyte. Usually the response time is measured to be less than a minute in the second range.[^39^, ^40^, ^41^, ^42^, ^43^] Similar to other reported sensors for organic substances our setup could be transferred to such compounds as long as a redox activity is found.

**Table 3:** Summary of the measured response times for all prepared IDE structures for platinum as well as carbon.

structure	material	Response time [s]
IDE1	platinum	9.8
IDE2	platinum	3.6
IDE4	platinum	22.6
IDE3C	carbon	11.3
IDE4C	carbon	17.9

## Conclusions

In this paper patterned micro sensors were printed in a wide range of line‐width from 500 nm to 100 μm with nanoimprint lithography and piezoelectric inkjet printing depending on the size. The structures, obtained from platinum as well as carbon precursors were used and electrochemically characterized by using an aqueous ferrocenyl‐methanol solution as model analyte. The limit of detection was determined to be as low as 35.9 μM for platinum and 1.2 μM for carbon structures. Furthermore, it was found that the carbon structures have an overall preferable performance in terms of LOD, sensitivity and response time. Crucial parameters that have influence on the performance of the structures seem to be a mix of diffusion length, surface area and the conductivity of the prepared structure. Response time was determined for both materials and methods where a minimum of 3.6 s for an intermediate structure size was found.

## Conflict of Interests

The authors declare no conflict of interest.

1

## Supporting information

As a service to our authors and readers, this journal provides supporting information supplied by the authors. Such materials are peer reviewed and may be re‐organized for online delivery, but are not copy‐edited or typeset. Technical support issues arising from supporting information (other than missing files) should be addressed to the authors.

Supporting Information

## Data Availability

The data that support the findings of this study are available from the corresponding author upon reasonable request.
