# Supplementary material for: Precursor‐Derived Sensing Interdigitated Electrode Microstructures Based on Platinum and Nano Porous Carbon
Source: ChemistryOpen. 2024 Aug 19;13(11):e202400179. doi: 10.1002/open.202400179 (PMC11564864; doi:10.1002/open.202400179)
Supplement: Supplementary file 1 — Supporting Information [file OPEN-13-e202400179-s001.pdf]

ChemistryOpen

Supporting Information

Precursor-Derived Sensing Interdigitated Electrode Microstructures Based on Platinum and Nano Porous Carbon

Lukas Mielewczyk, Lydia Galle, Nick Niese, Julia Grothe,* and Stefan Kaskel

Supporting Information

Precursor-Derived Sensing Interdigitated Electrode Microstructures Based on Platinum and Nanoporous Carbon

Lukas Mielewczyk, Lydia Galle, Nick Niese, Julia Grothe, Stefan Kaskel*

Department of Inorganic Chemistry, Technische Universität Dresden, Bergstrasse 66, 01069 Dresden, Germany

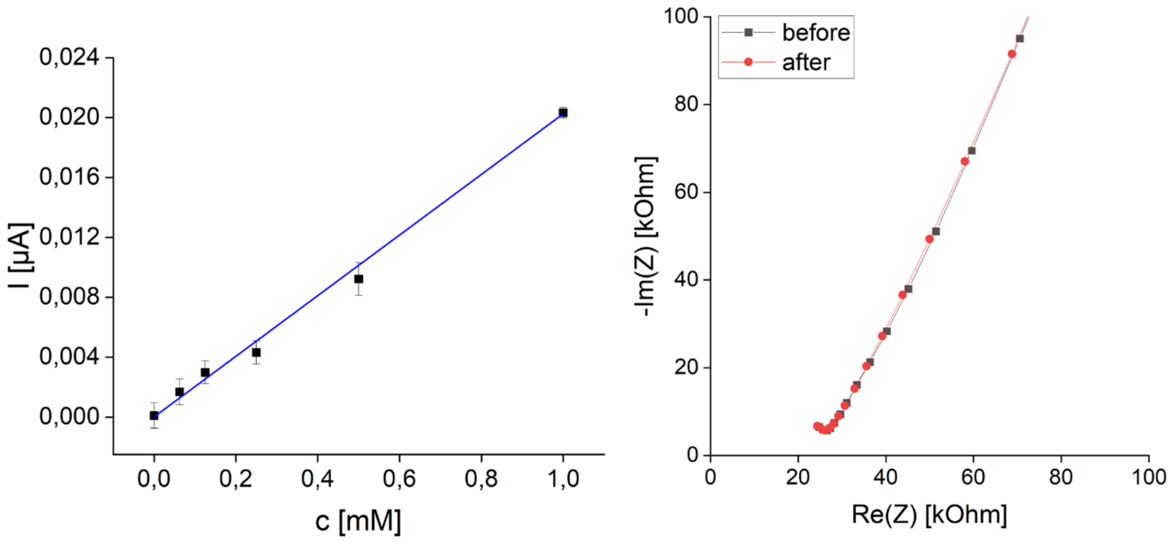

Figure S1. (left) CA of IDE1 platinum with linear fit, (right) PEIS measurement of IDE1 platinum before (black) and after(red) cyclovoltammetry.

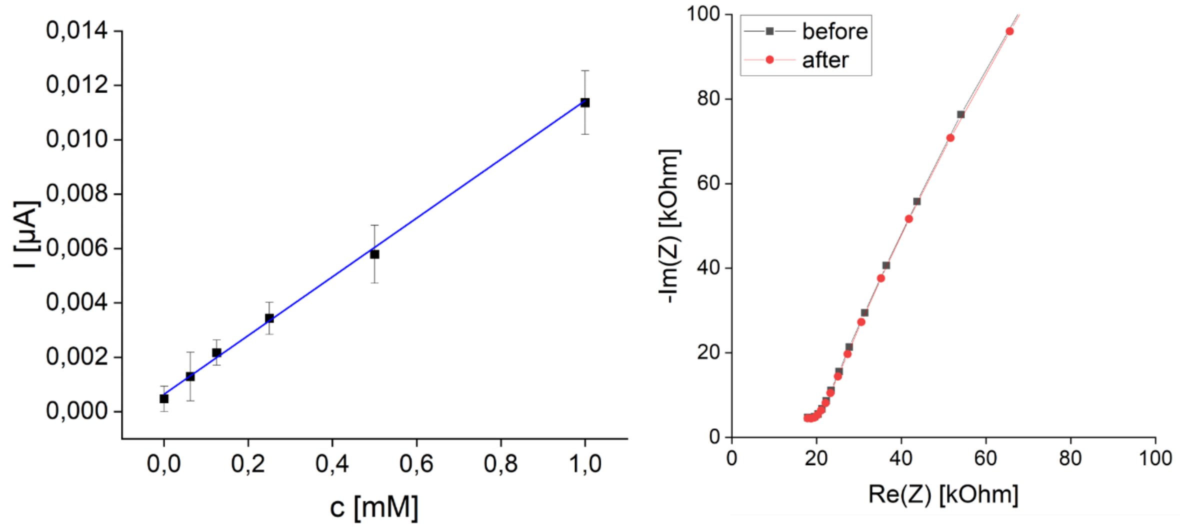

Figure S2. (left) CA of IDE2 platinum with linear fit, (right) PEIS measurement of IDE2 platinum before (black) and after(red) cyclovoltammetry.

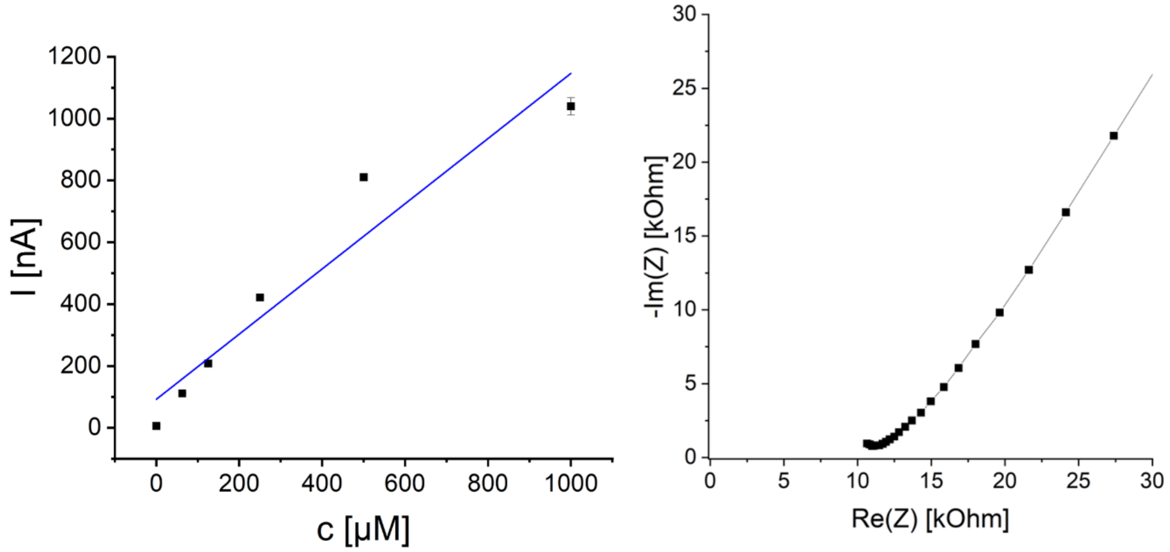

Figure S3. (left) CA of IDE3 platinum with linear fit, (right) PEIS measurement of IDE3 platinum.

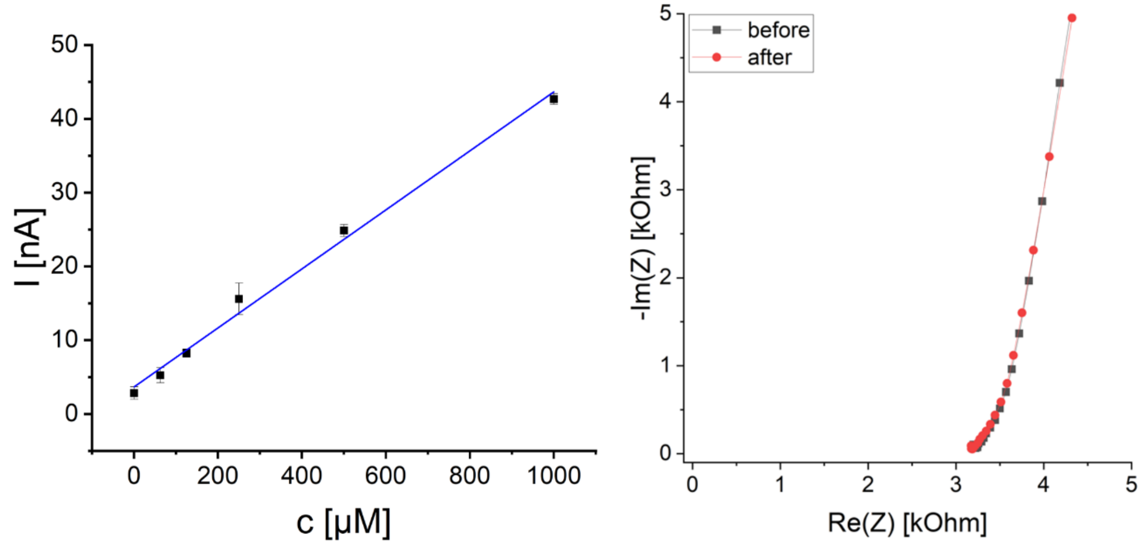

Figure S4. (left) CA of IDE3 carbon with linear fit, (right) PEIS measurement of IDE3 carbon before (black) and after(red) cyclovoltammetry.

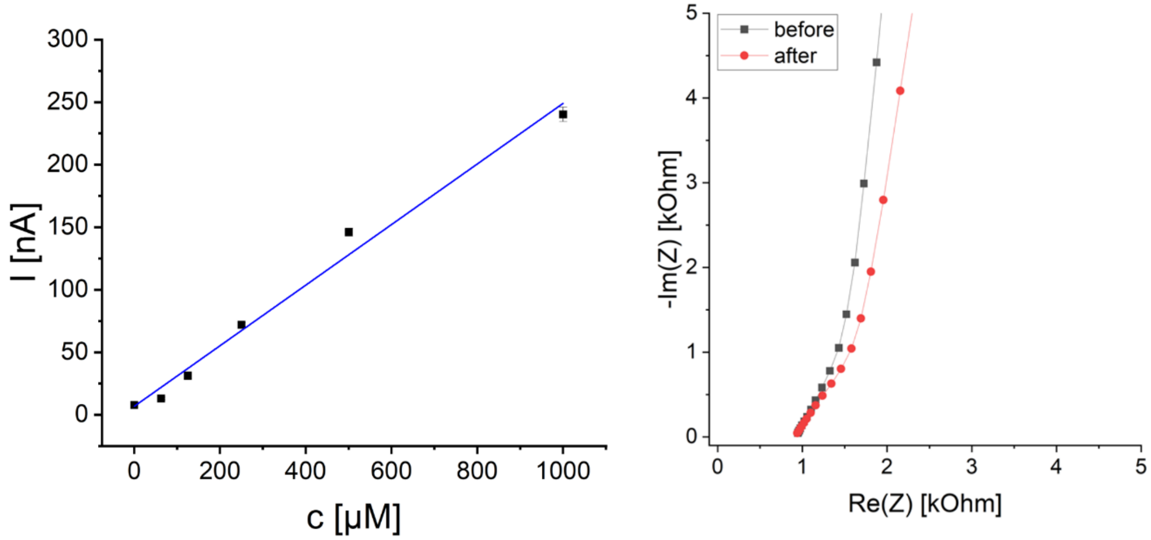

Figure S5. (left) CA of IDE4 carbon with linear fit, (right) PEIS measurement of IDE4 carbon before (black) and after(red) cyclovoltammetry.
